# Connectivity from spike trains of neocortex neuron populations

**DOI:** 10.1186/1471-2202-15-S1-P18

**Published:** 2014-07-21

**Authors:** Mark Hereld, Jyothsna Suresh, Mihailo Radojicic, Lorenzo L Pesce, Wim van Drongelen

**Affiliations:** 1 Computation Institute, The University of Chicago and Argonne National Laboratory, IL, USA; 2 Mathematics and Computer Science, Argonne National Laboratory, Argonne, IL, USA; 3 Department of Pediatrics, The University of Chicago, Chicago, IL, USA

## 

A principal goal of this work is to determine connectivity in cultured neuronal networks as they mature so that we can understand the relationship between connectivity and behavior in biologically generated neuronal networks. A challenge of this aim is that we are able to measure only a relatively small number of neurons. In our lab the cultures are grown on a arrays of 60 electrodes. With current techniques we can distinguish at most a handful of spiking neurons while mature cultures comprise more than 10K neurons. At this stage in the work we have collected data during development of cultures over a full 30 minutes of sub-millisecond samples.

In this paper we present two complementary approaches to the problem. First, we present our results of a brute force search for precisely matched timing differences between events in a detailed 30-minute recording of events from one of our cultures. Here, we are attempting to identify candidates for membership in polychronous groups [1] of neurons that have been triggered at least twice within the experimental window. Our second approach is to simulate populations of neural networks wherein the connectivity is completely known. From simulations of this system we analyze analogs to the subset of experimentally detected events in order to better understand how much of the underlying connectivity might be available to us through such a limited window.

With the polychronous group model as our guide we focus our search on instances of fixed time differences between sets of events on electrode pairs, indicating a set of neurons that are part of the same group. In a brute force search through the spike data we see significant departures from chance occurrence of repeated intervals in spikes from pairs of channels. One 30-minute recording included over 50 thousand spike events on 9 electrodes. Analyzing the 5109 spike events found outside of dense bursts, we identified 91 K occurrences of identical interval instances on an electrode pair. For example, in our initial study of two small peaks in the histogram of spike intervals, the observed number of repeats in each peak would have been expected in fewer than 3 out of 1000 such peaks if the spikes in the involved channels were uncorrelated.

In order to understand the limitations that such a measurement system faces, we also study artificial neural networks with known connectivity. To this end we perform computer simulations wherein we vary the amount and character of information available to the analysis (number of electrodes and neurons per electrode) to test the relationship between the number of measurable neurons and the quality and character of inferred connectivity. We study one approach to winnow the search space by collecting data at intervals during the development of the network to include early stages when there are relatively few connections, and therefore relatively few gaps in our knowledge. Inferences about early stage connectivity could be used to constrain possible networks at later stages. We test this hypothesis computationally and report our results.
